# Underwater Object Recognition Using Point-Features, Bayesian Estimation and Semantic Information

**DOI:** 10.3390/s21051807

**Published:** 2021-03-05

**Authors:** Khadidja Himri, Pere Ridao, Nuno Gracias

**Affiliations:** Underwater Robotics Research Center (CIRS), Computer Vision and Robotics Institute (VICOROB), University of Girona, Parc Científic i Tecnològic UdG C/Pic de Peguera 13, 17003 Girona, Spain

**Keywords:** 3D object recognition, point clouds, global descriptors, semantic segmentation, semantic information, Bayesian probabilities, laser scanner, underwater environment, pipeline detection, inspection, maintenance and repair, AUV, autonomous manipulation, multi-object tracking, JCBB

## Abstract

This paper proposes a 3D object recognition method for non-coloured point clouds using point features. The method is intended for application scenarios such as Inspection, Maintenance and Repair (IMR) of industrial sub-sea structures composed of pipes and connecting objects (such as valves, elbows and R-Tee connectors). The recognition algorithm uses a database of partial views of the objects, stored as point clouds, which is available *a priori*. The recognition pipeline has 5 stages: (1) Plane segmentation, (2) Pipe detection, (3) Semantic Object-segmentation and detection, (4) Feature based Object Recognition and (5) Bayesian estimation. To apply the Bayesian estimation, an object tracking method based on a new Interdistance Joint Compatibility Branch and Bound (IJCBB) algorithm is proposed. The paper studies the recognition performance depending on: (1) the point feature descriptor used, (2) the use (or not) of Bayesian estimation and (3) the inclusion of semantic information about the objects connections. The methods are tested using an experimental dataset containing laser scans and Autonomous Underwater Vehicle (AUV) navigation data. The best results are obtained using the Clustered Viewpoint Feature Histogram (CVFH) descriptor, achieving recognition rates of 51.2%, 68.6% and 90%, respectively, clearly showing the advantages of using the Bayesian estimation (18% increase) and the inclusion of semantic information (21% further increase).

## 1. Introduction

With the recent developments in the robotics industry there has been an increasing use of vehicle-mounted sensors. These sensors seek to provide useful information to the user, such as a clear perception of the environment, or provide more specific details such as obstacles to be avoided or objects to interact with. The outputs of these different sensors lead to different representations of the environment, depending on the sensor used and the task to be accomplished.

Previous work on methods for collecting and interpreting spatial data for mobile robotics could be broadly divided into into three main categories. The first focuses prominently on data providing a 2D representation of the environment, such as images from cameras. The second relies on 3D point cloud data from sensors like laser scanners or acoustic ranging. The third uses hybrid data, either combining data from two different sensors or using a composite sensor such as the Microsoft Kinect that provides both images and point clouds. Over the last decade 3D point clouds have been widely used in computer vision and mobile robotics applications, opening the door to important but challenging tasks such as 3D object recognition [1,2,3,4,5,6] and semantic segmentation [7,8,9], which are core steps for scene understanding.

Understanding scenes and being able to navigate while detecting objects of interest is a fundamental task for self-driving vehicles and autonomous robots. To navigate an environment, the robot needs to build a representation of the content of the scene that encapsulates the location of objects of interest within the environment.

In this line of research, the combined use of 3D object recognition and semantics has contributed to the development of better approaches to scene understanding. In the last decade various methods based on point clouds have been proposed, aiming to solve semantic segmentation. Semantic segmentation [10,11,12] can be broadly defined as the task of grouping parts of the input data, which can be 2D or 3D images or even 3D point clouds, which belong to the same object class, thus classifying each pixel or 3D point in the input according to a category.

Most of the recent methods deploy deep learning techniques while considering object models as black boxes. This trend is highlighted in the survey published by Guo et al. [13] on recent work on deep learning methods for point clouds, including semantic segmentation. Their survey reviews the most relevant applications for point cloud understanding, within the topics of 3D shape classification, 3D object detection and tracking and 3D point cloud segmentation. A review of state-of-the-art Deep Learning methods is presented using various publicly available datasets.

Semantic segmentation was inspired by the success of Deep Learning methods in producing an accurate result [10,13,14], but these techniques require an extremely large amount of data to train the network. Such large datasets may be difficult to obtain, or not provide adequate information, such as the case of man-made structures captured by sensors that only provide colourless point clouds.

3D object recognition based on point clouds has been studied across various disciplines, with an emphasis on deep neural network based approaches and feature point based methods. Relevant research in this area has been summarized and organized in various survey, using global and local methods [3,15]. Global recognition methods describe the entire object as a single vector of values, whereas local recognition methods are more focused on local regions and are only based on salient points.

Accurate and efficient algorithms for segmentation and recognition are required for the emerging Inspection, Maintenance and Repair (IMR) applications, especially given the recent advances in laser scanning technology. An example of critical application scenarios, that are attracting increasing research interest, are construction sites such as refineries which have extensive networks of industrial pipelines, that need frequent inspection and intervention.

Research in segmentation and recognition for pipeline sites has been conducted by Huang et al. [16] and Pang et al. [17], where a complex pipeline structure is partitioned and modeled as a set of interconnected parts using a Support Vector Machine (SVM)-based approach and a single local feature descriptor. Another notable application to pipeline classification is the work of Kumar et al. [18], in which an aerial vehicle equipped with a low-cost Light Detection and Ranging (LIDAR) is able to map and identify pipes of different lengths and radii. Ramon et al. [19] proposed a visual algorithm based on a semantic Convolutional Neural Networks (CNN) to detect pipes. The authors presented an approach based on a drone capable of autonomously landing on pipes, for inspection and maintenance in industrial environments. More recently, Kim et al. [20] presented an automatic pipe-elbow detection system in which pipes and elbows were recognized directly from laser-scanned points. The methods they used are based on curvature information and CNN-based primitive classification.

Regarding marine applications, the use of vision sensors underwater is becoming widespread. However, these sensors impose strong requirements related to water turbidity and the presence of light, to capture high quality images. Since the underwater images are subjected to rapid attenuation and scattering of light, object detection and recognition can only be performed at very short distances from objects, of the order of a few meters. Acoustic propagation allows much longer ranges in terms of sensing distance, but the object representations obtained are much too noisy and coarse in resolution to allow accurate object identification and localization for autonomous object grasping.

A comparatively small number of object recognition applications have been reported underwater. These include pipeline identification and inspections based on optical images in seabed survey operations [21], cable identification and pipeline tracking based on acoustic images [22] and recognition of different geometric shapes such as cylinders and cubes [23] using acoustic imaging cameras.

Similarly to in-air applications, Deep Learning methods have been quickly adapted to handle object recognition in underwater environments. In [24], Yang et al. applied both YOLOv3 [25] and Fast Region-based Convolutional Network (Faster R-CNN) [26] methods based on deep learning to localise and classify the images from their dataset *Underwater Robot Picking Contest (URPC)* into three categories—sea cucumber, sea urchin and scallop. The two algorithms were used in comparative experiments, to select the best algorithm and model for target detection and recognition, as part of their underwater detection robot. In [27], a detailed review of Deep Learning-based object recognition is given, whether it is underwater or surface target recognition. Surface object recognition is mostly based on images, while underwater objects are recognized based on videos, target radiated noises [28] and acoustic noises [29]. While Deep Learning outperforms traditional machine-learning methods when large amounts of training data are available, it also imposes additional effort in the annotation of those large amounts of data. Work on detection and mapping of pipelines and related objects has focused, almost exclusively, on above-water scenarios. An exception is the work of Martin et al. [30], which presents an approach based on a deep neural network PointNet [31]. These authors are able to detect pipes and valves from 3D point clouds with RGB color information, obtained with a stereo camera, using their own dataset to train and test the network.

### 1.1. Objectives and Contributions

The present paper develops a semantic Bayesian model for the recognition of 3D underwater pipeline structures. The proposed approach builds upon our previous work in [3], and extends it in several directions. The present work was motivated by the challenges stemming from real data collected under realistic underwater conditions with an AUV equipped with a fast laser scanner developed at our research center [32]. An example of the challenging conditions is the fact that data is collected by a free-floating, platform whose movements create deformations of the perceived shape of the objects which are difficult to be corrected with the typically available sensors, such as Inertial Measurement Unit (IMU) and Doppler Velocity Log (DVL). Three main contributions of the present paper can be summarized as follows.

- The 3D complexity of pipeline structures makes segmentation a difficult issue to deal with. Our test structure, which is described in further detail in Section 5, includes four different types of objects: two different valves (*Butterfly-Valve* and *Ball-Valves*), an *Elbow* and a *r-R-Tee*. These objects are connected by cylindrical pipes. In this paper a semantic segmentation method is proposed, based on geometric constraints together with rules for decomposing connected pipe structures. The aim of this method is to separate and distinguish, at the point cloud level, the points that belong to objects and those that belong to connecting pipes.
- Most global 3D descriptor methods assume that the point clouds are de-noised, complete, and consistent. This is not always the case, specially for the conditions that we are targeting in this paper, where the objects may be partially occluded due to the cluttered nature of the pipelines, and the point clouds may be inconsistent due to un-modeled deformations caused scanner motions during acquisition. These conditions commonly lead to false detection and overall failure of the global descriptor methods. Additionally, the similarity between objects can also lead to confusion when only a small or non-informative part of the object is observed. To overcome these limitations, a Bayesian semantic model is proposed. Taking advantage of the results obtained in our previous work [3], a confusion matrix was created for different global descriptors and objects. In this study, only the two best performing descriptors were considered: CVFH and Oriented, Unique and Repeatable (OUR-CVFH).
- To feed the Bayesian estimation model, observations of the same object across multiple scans are required. However, the underwater data suffer from the lack of DVL tracking during the descent of the AUV and sometimes during the mission when, for example, the sensor beams touch the side slopes of the test tank facility. The loss of DVL tracking leads to a rapid degradation of the estimates of the absolute pose of the pipeline structure with respect to the vehicle, which in turn hinders the ability to correctly perform the tracking of the objects. To overcome this problem, a multi-object tracking method inspired in the Joint Compatibility Branch and Bound (JCBB) algorithm [33] was proposed.

### 1.2. Structure of the Paper

The remainder of the paper is organized as follows. Section 2 describes the processing pipeline that is proposed in this paper. It includes a description of the object database, the algorithms used for pipe detection and semantic object detection, and the object recognition based on global descriptors. Section 3 describes the Bayesian Recognition component of our approach. It details the object tracking and Bayesian estimation processes. In Section 4, the algorithm developed for the recognition based on semantic information is detailed. Section 5 presents a description of the experimental hardware, the testing conditions and the analysis of the experimental results. This analysis is separated in terms of average and class-by-class performance, followed by a discussion of results. Finally Section 6 and Section 7 present the overall conclusions of this work and lines for further research, respectively.

## 2. 3D Object Recognition Pipeline

**Figure 1:**
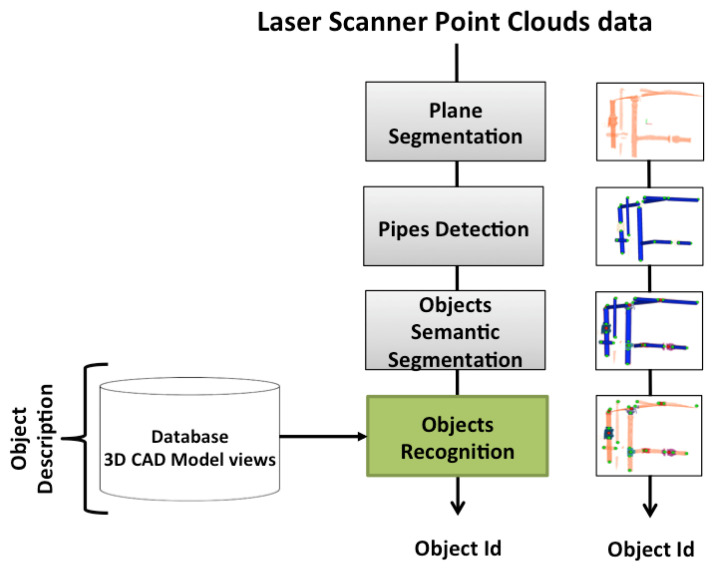
3D Object Recognition Pipeline.

Our recognition strategy focuses on object recognition of connected objects, which includes polyvinylchloride (PVC) pipes and attached elements, such as simple pipe connectors and valves suitable for manipulation and intervention. The proposed recognition pipeline is shown in Figure 1. The method uses, as input, a 3D point cloud acquired by a laser scanner mounted on an AUV. The scene contains objects for which 3D models are available *a priori* in a database. These objects are interconnected through pipes. The goal of the algorithm is to identify these objects by returning the class of the object with its associated Bayesian probability.

As shown in Figure 1, the recognition pipeline is divided into different modules described in the following subsections.

### 2.1. Object Data Base

**Figure 2:**
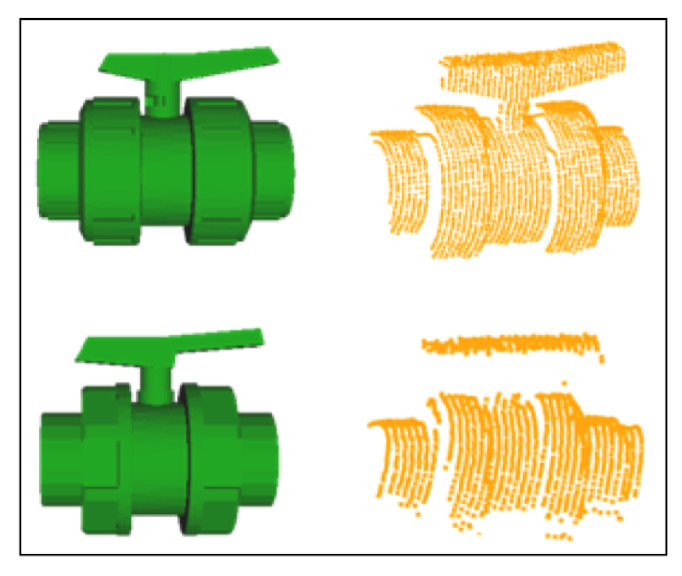
Ball-valve (**top**) and Ball-valve-s (**bottom**) with their respective segmented scan.

The data base contains 3D models of the *a priori* known objects. Each one is modelled as a set of overlapping partial views stored as point clouds and covering the full object. The details on how the data base was built are presented in [3]. The only difference regarding the database used in the present paper is that, given the similarities of the partial views of *Ball-Valve* and the *Ball-Valve-S* (as can be seen in Figure 2) it was decided to merge these two classes into a single class labelled *Ball-Valve*.

The most relevant characteristics of the objects in the database are illustrated in Table 1 including their views.

### 2.2. Plane Segmentation

Our recognition system was tested in a robotics testing pool, as described in Section 5. The pool walls appear in the scans as large co-planar sets of points. These surfaces need to be removed in order to avoid unnecessary interference with the semantic segmentation that will be applied to the industrial pipe structure. In order to achieve this, a plane segmentation procedure was implemented using the Random Sample Consensus (RANSAC) [34] algorithm already available in Point Cloud Library (PCL) [35]. Due to the fact that the AUV is free-floating and moving during the scan acquisitions, the sets of points corresponding to the pool walls are not precisely co-planar. In fact, they follow a slightly curved but almost flat surface, which is not straightforward to describe parametrically. However good results for the plane extraction can be achieved by properly adjusting the acceptance threshold in the plane-fitting algorithm.

### 2.3. Pipe Detection

The next step is to detect the pipes that are visible within the current scan. A variety of methods exist to estimate the parameters of primitive geometric shapes such as planes, spheres, cylinders, cones, within 3-D point clouds [36,37,38,39,40]. In our case, a method based on RANSAC-PCL [35] has been applied to detect the pipes in the scene which are modelled as as cylinders of similar radii. The RANSAC-PCL method uses a seven parameter description of the cylinders, where the first three represent a point on the axis, the second three represent the direction of the axis, and the last one represents the radius of the cylinder. Since the diameters of the pipes are known and equal to 0.064 m, we look for potential candidate cylinders whose radii are within a tolerance of this value.

**Figure 3:**
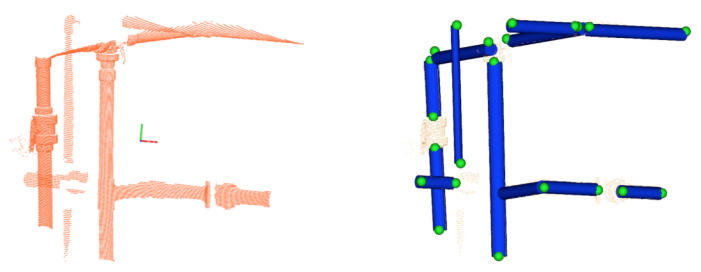
Pipes detection: (**left**) 3D laser scan point cloud; (**right**) pipes with their respective endpoints.

**Figure 4:**
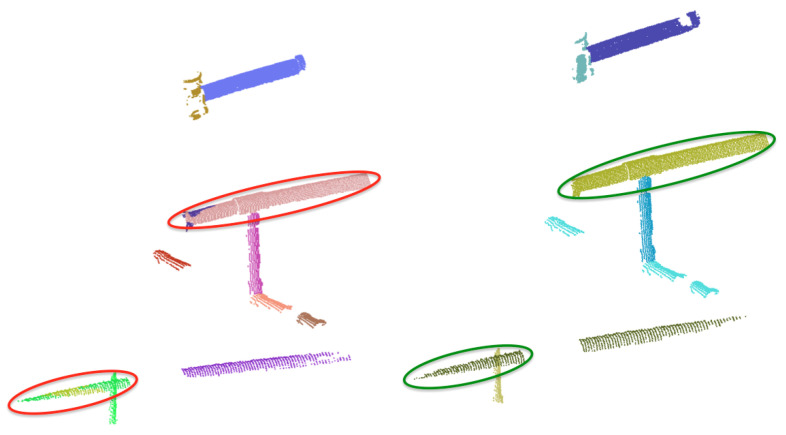
Pipes Merging: (**left**) Pipe detection result previous to merging showing, within circles, multiple pipe detections of the same pipe; (**right**) Result after merging where the multiple detections have been merged into a single one.

Once the set of points belonging to a cylinder has been identified, the location of the extremities and the length can be computed by projecting the points on the cylinder axis and calculating the maximum and minimum of the segment defined by the projection. Figure 3 shows, for a given scan, all detected pipes with their respective endpoints. Unfortunately, in some cases, the same pipe may generate two different cylindrical point clouds. As shown in the encircled area of the left Figure 4, two pipes where detected, one appearing in red (the long one) and the other in blue (small section of a pipe). This happens due to small deformations of the scan caused by the motion induced distortion present in the underwater laser scanner [41]. Therefore, it is necessary to identify and fuse the point clouds that correspond to the same pipe segment (Algorithm 1) in order to provide a set of non duplicated pipes as input to the next module. The right side of Figure 4, shows the result after the merging.
**Algorithm 1:** Detection of Pipes and Extremities
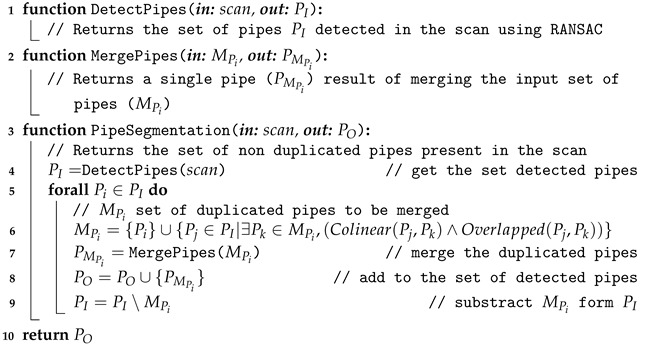


### 2.4. Semantic Object-Segmentation

This block of the procedure handles segmenting the object point clouds, from an input scan, containing pipes and objects. Instance segmentation is the process of clustering of input data (e.g., image or point cloud) into multiple contiguous parts without regard to understanding the context of its environment. One of the drawbacks of instance segmentation is that it relies on object detection methods to find the individual instances, which results in segmenting only the detected instances, so its performance in terms of over- or under-segmentation, depends on the result of the object detection method used.

**Figure 5:**
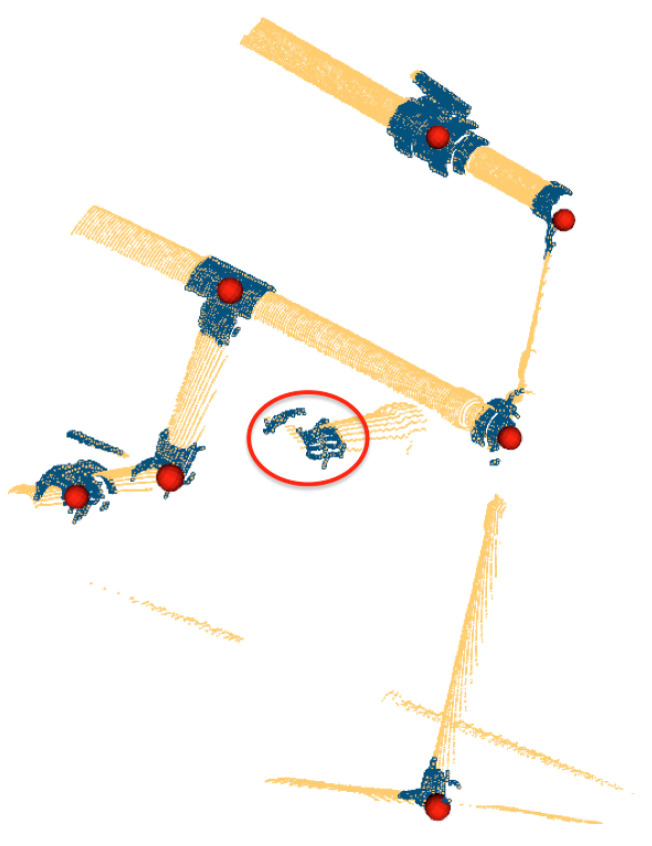
Semantic Segmentation: Red points represent the centroids of segmented objects. The red circle shows a segmented object located at an isolated extremity.

By contrast, semantic segmentation partitions the scenes into semantically meaningful parts, based on the understanding of what these parts represent, classifying each part into one of the pre-determined classes: pipes and objects. Therefore, semantic segmentation can be used to segment point clouds corresponding to challenging scenes where objects are connected to pipes. Since the pipes have been already detected, and because they are connected through objects, it is possible to exploit the connectivity and pipe intersections to guide the segmentation process. The SemanticSegmentation(·) (Algorithm 2) is organized in 4 steps:

1. Compute pipe intersections: This is done by the *Connected* function (Line 1) which, for each pair of pipes, checks if they are connected through an object and returns the pipe intersection point. To be connected, the axes of both pipes should be co-planar and two of their extremities should be close enough. By close enough, we consider that their distance should be smaller than the object size. Ideally, co-planarity means that the axes, when taken as infinite lines, will intersect. In reality, the axis lines estimated for the two pipes may not intersect, but will have a small distance between them. Therefore co-planarity is assessed by checking the inter-line distance.
2. Compute candidate object locations at the intersections: Each pair of pipes defines an ’intersection’ point. Therefore, if we have 3 pipes connected to an object (e.g., the *R-Tee*), we have 3 pairs of 2 pipes having, therefore, 3 intersection points. The function *ComputeIntersectionLocations* in line 16 clusters the intersection points corresponding to the same object and computes their centroids, to obtain a single location for each object.
3. Compute candidate object locations at isolated pipe extremities: Because of the iterative nature of the scanning process it may happen that a pipe appears in a scan together with an object at its extremity, while the other pipes connected to the object have not yet been detected. The function *ComputeExtremityLocations* in line 17 computes the object locations in these cases. The outcome of this step is shown in Figure 5.
4. Crop the objects from the input scan: Once the object locations are known ($C_{i}\cup C_{e}$), and knowing the dimensions of the objects, the points contained in a predefined bounding box are cropped (line 25) and returned for object recognition.

**Figure 6:**
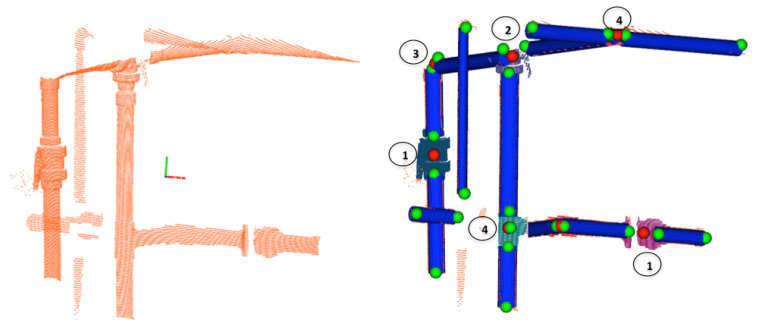
Semantic Segmentation: (**Left**) Input 3D point cloud; (**Right**) Pipes (blue cylinders) with their endpoints (green spheres), and the centroids of the objects to be segmented (red spheres) along with the segmented objects point clouds (colored). The objects 1, 2, 3, 4 represent respectively: a *Ball-Valve*, a *3-Way-Valve*, an *Elbow* and a *R-Tee*.

Figure 6 shows an example of semantic segmentation where the candidate object locations can be appreciated together with the segmented point clouds.
**Algorithm 2:** Semantic Segmentation
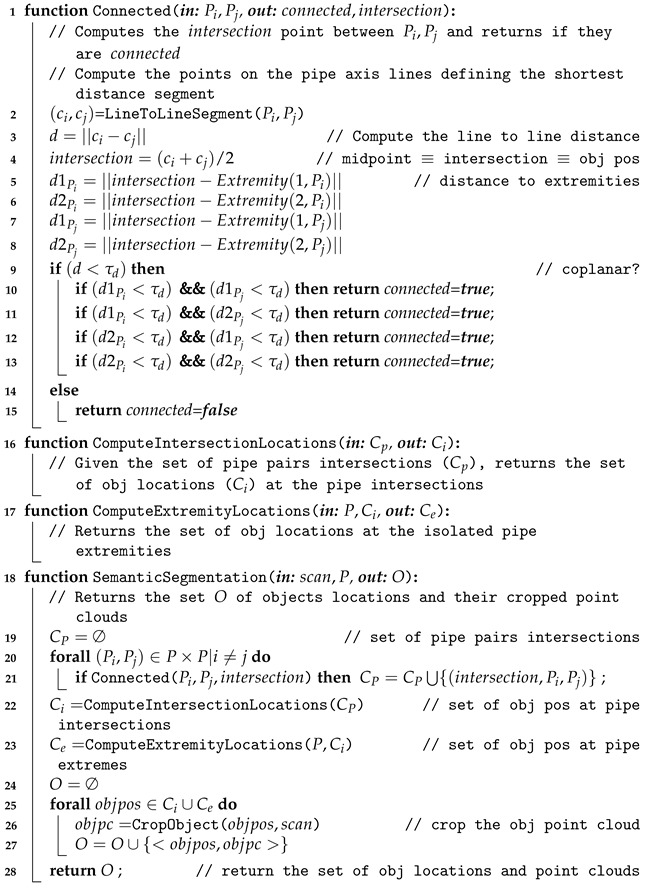


### 2.5. 3D Object Recognition Based on Global Descriptors

Object recognition is based on the use of the global descriptors that we studied and compared in [3]. The Clustered Viewpoint Feature Histogram (CVFH) [42] and the Oriented, Unique and Repeatable CVFH (OUR-CVFH) [43] were the two descriptors that achieved the best overall performance, so we have selected only these two descriptors. A summary of their characteristics is presented in Table 2.

The descriptors are used to encode, in a compact way, the objects segmented in the previous step. They also encode the object views stored in the database (see Table 1). In this way, the segmented objects can be matched against the model views, comparing the segmented input scan, with all the views of the object models in the database. Using the chi-square distance, as proposed in [44,45], the database view corresponding to the smallest distance is selected.

## 3. Bayesian Recognition

One of the problems of performing single view object recognition as proposed above (in Section 2.5) is that several objects may have similar views. Partial views of the *R-Tee* may be easily confused with the *Elbow* for instance. In [3] we studied the confusion matrices for the different objects. The confusion matrices state, for *n* observations of a given object, how many of them were recognised as *object-class-1*, how many as *object-class-2* and so on. Therefore, they can be easily converted into probabilities which can be used to implement a Bayesian estimation method for object recognition to attain more robust results. This is achieved by combining several observations to compute the probability that an object belongs to each object-class, selecting, then, the one with highest probability as the solution. To do this, first it is necessary to be able to track the objects across the scans (as described in Section 3.1) so that their Bayesian probabilities can be iteratively computed (Section 3.2).

### 3.1. Object Tracking

**Figure 7:**
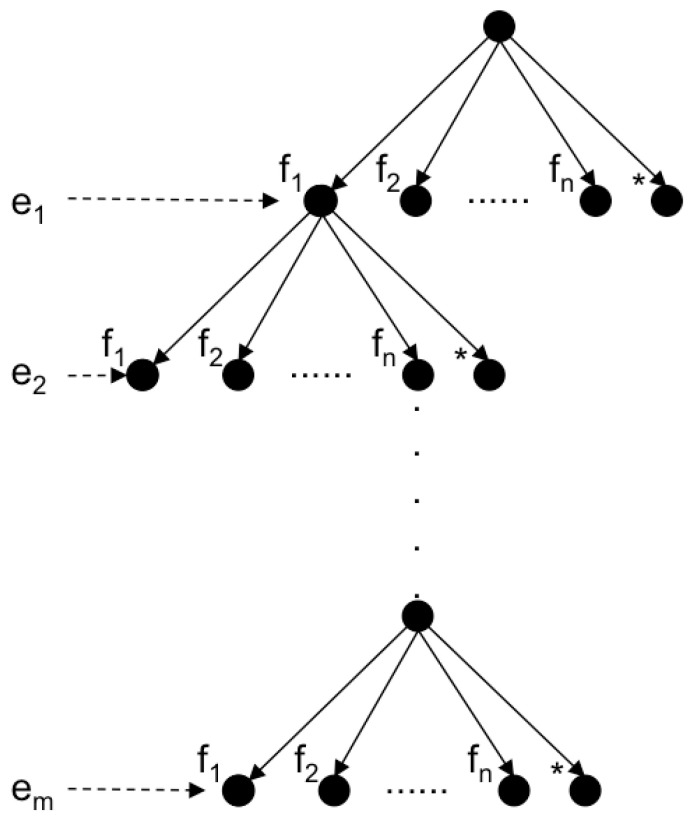
Interpretation tree stating, for each object *e_i_* (level *i*) its potential associations *f*_1…*n*_, representing the (*) node, a spurious measurement.

To track objects across the scans we have to solve the data association problem. The simplest way to do it is to use the Individual Compatibility Nearest Neighbour (ICNN). This can be done if a reasonable dead reckoning navigation is available. In presence of significant uncertainty ICNN is not enough, and more powerful strategies such as the JCBB [33] are required. JCBB explores the interpretation tree (Figure 7) searching for the hypothesis with largest number of jointly consistent pairings between measurements ($e_{i}$) and features ($f_{j}$). The validation of the hypothesis is based on two conditions: (1) the candidate set of pairings must be individually and jointly compatible and (2) only those hypotheses that may increase the current number of pairings are explored (bound condition). The first condition is achieved by comparing the Mahalanobis distance of the set of candidate pairings with a threshold, defined at a given confidence level, of the related Chi-square distribution. The second condition is met by estimating the maximum number of pairings we can achieve if we keep exploring the current branch. Since each depth level of the tree represents a potential pairing, the number of levels below the node of the current hypothesis is an estimate of the maximum number of pairings we may add by exploring the current branch. Then it is only worth continuing exploring if the number of pairings of the current hypothesis plus the maximum number of achievable pairings is higher than the one associated with the current best hypothesis.

Unfortunately, when an AUV navigates close to vertical 3D structures, like a water tank, some DVL beams may suffer from multi-path effects leading to incorrect localization (position jumps). This type of error cannot be solved using the standard JCBB. For this reason, a navigation-less variation of the JCBB algorithm based on the intra-scan inter-object distances is proposed in Algorithm 3, which will be referred to as IJCBB. In this case, the algorithm pairs objects that are present in two scans so that all their inter-distances in both scans remain unaltered. Let us consider two sets of object locations $E=\{e_{1},\cdots ,e_{m}\}$ and $F=\{f_{1},\cdots ,f_{n}\}$ segmented from two given scans ($S_{E}$ and $S_{F}$), whose objects we want to associate. A matching hypothesis is defined as a set of non-duplicated potential pairings from both scans:

(1) $$H=\{p_{ij}=\left(e_{i},f_{j}\right)\in E\times F/\forall p_{kl}\in H\Rightarrow i\neq k\;and\;j\neq l\}.$$

An hypothesis is considered to be jointly compatible if and only if, the distance between any two objects in scan $S_{i}$ and the corresponding distance of their matching objects in scan $S_{j}$ also matches:

(2) $$H\;\mathrm{Jointly}\;\mathrm{compatible}\Leftrightarrow (\forall \;p_{ij},p_{kl}\in H\Rightarrow ||e_{i}-e_{k}||=||f_{j}-f_{l}||).$$

**Figure 8:**
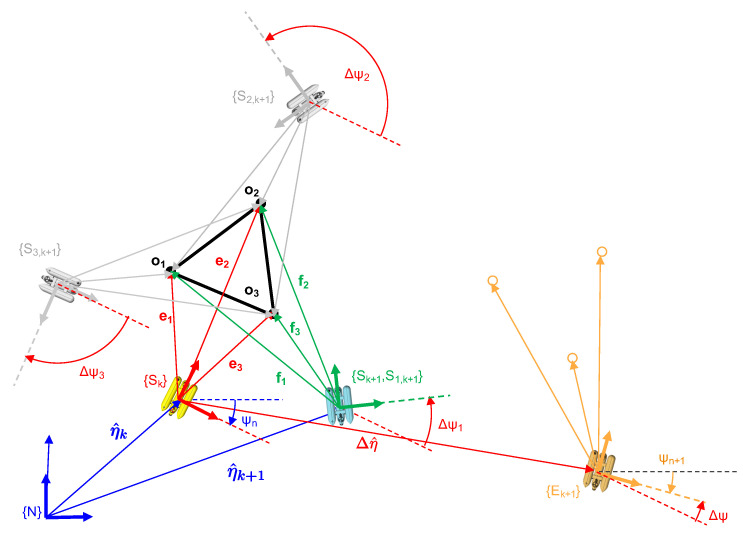
Roto translation estimation.

Then, as stated above, the goal of IJCBB (Algorithm 3) is to find the largest hypothesis $H_{L}$ for which the condition in Equation (2) holds. Once $H_{L}$ has been computed, the roto-translation transformation between both scans can be computed using Single Value Decomposition (SVD) [47]. The minimum number of matching pairs required to solve for the roto-translation is 3, which defines 3 inter-distances. Figure 8 shows an example of the ambiguities that may arise using 3 pairs only.

Let us consider a robot located at a pose $\eta_{k}$ (yellow) moving, during a small time interval Δt, a displacement Δη to achieve a new pose $\eta_{k+1}$ (green). Let ${\hat{\eta }}_{k}$, $\Delta \hat{\eta }$ and ${\hat{\eta }}_{k+1}$ be the estimates of the corresponding vectors. If $\Delta \hat{\eta }$ is incorrect due to a failure in the navigation sensors, the estimated robot location at time k+1 (${\hat{\eta }}_{k+1}$) is also erroneous (frame $\left\{E_{k+1}\right\}$ in orange). Now, let us consider 3 equidistant objects: $o_{1}$, $o_{2}$ and $o_{3}$, observed from $\left\{S_{k}\right\}$ as: $e_{1}$, $e_{2}$ and $e_{3}$ as well as from $\left\{S_{k+1}\right\}$ as: $f_{1}$, $f_{2}$ and $f_{3}$. Since the 3 inter-distances are equal, 6 possible pairings exist ($\left\{e_{1}f_{1},e_{2}f_{2},e_{3}f_{3}\right\}$, $\left\{e_{1}f_{2},e_{2}f_{3},e_{3}f_{1}\right\}$, $\left\{e_{1}f_{3},e_{2}f_{1},e_{3}f_{2}\right\}$, $\left\{e_{1}f_{3},e_{2}f_{2},e_{3}f_{1}\right\}$, $\left\{e_{1}f_{1},e_{2}f_{3},e_{3}f_{2}\right\}$, $\left\{e_{1}f_{2},e_{2}f_{1},e_{3}f_{3}\right\}$), the first 3 (the ones involving a rotation in the plane only) are shown in Figure 8. The other three are not considered since they involve a motion (in pitch) which the robot cannot manage. The actual solution corresponds to frame $\left\{S_{1,k+1}\right\}$ (in green) while the others ($\left\{S_{2,k+1}\right\}$ and $\left\{S_{3,k+1}\right\}$ both in grey) are not correct. Given the fact that we are tracking the robot pose, Δt is very small so the smallest motion (lower $\Delta \psi_{i}$) can be considered the correct one. In case only two inter-distances are equal, then four pairings exist and only two are relevant. Again, the smallest motion heuristic can be applied. When all the inter-distances are different a single pairing exists.
**Algorithm 3:** IJCBB
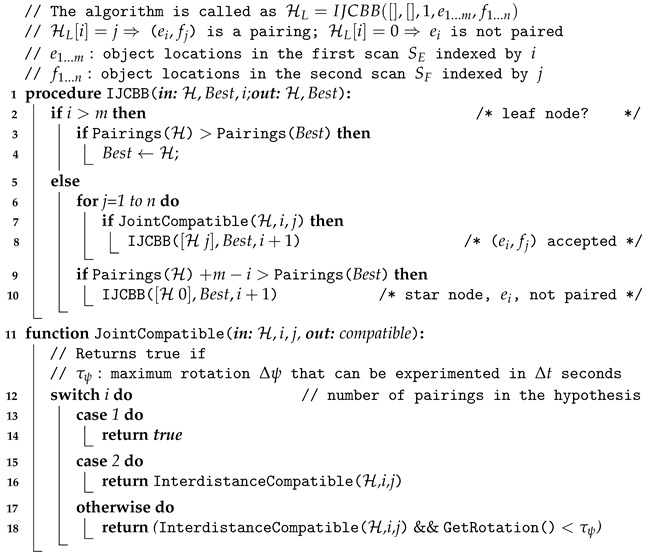


It may also happen that Equation (2) holds for an incorrect data-association hypothesis. This means that we can have two different sets of objects, having the same inter-distances. This may happen when scanning repetitive structures, for instance. Again, because Δt is small, the small motion heuristic also works providing the correct roto-translation. For these reasons, the JointCompatible(·) function in Algorithm 3 checks the rotation angle implied by the hypothesis H, which should be small enough to be considered jointly compatible.

**Figure 9:**
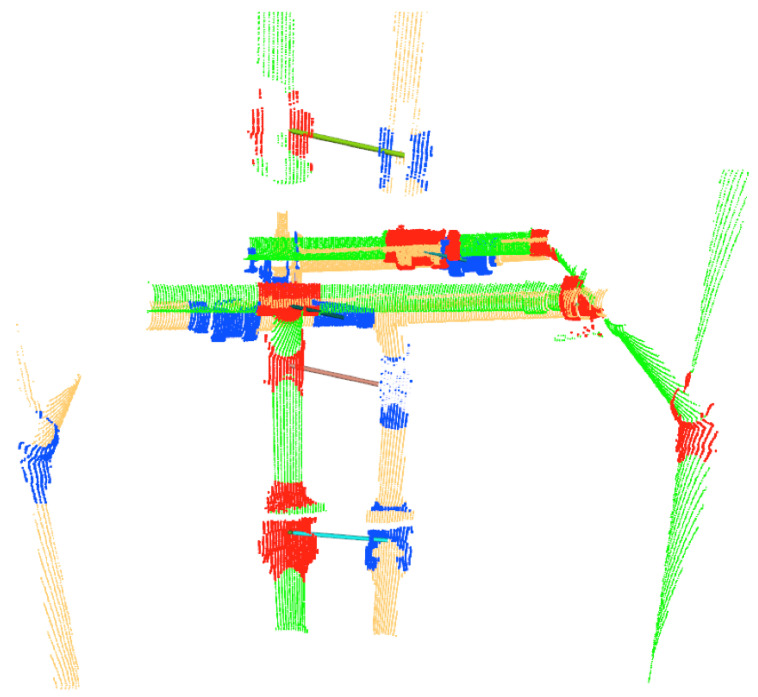
Tracking objects over two consecutive scans, represented in green/red and yellow/blue. The significant displacement between the two scans is the results of navigation inaccuracies from noisy Doppler Velocity Log (DVL) readings in the test pool. The solid lines indicate the objects associated by the tracking.

Figure 9 shows the tracking of two consecutive scans using IJCBB. The red objects were detected from $S_{E}$ and the blue ones from $S_{F}$, corresponding to the previous and the current scan. In this case five objects were paired, while other three were discarded.

### 3.2. Bayesian Estimation

The objects can often be confused with others. This happens because we are dealing with partial views of the objects appearing in the scans, which may match several views of other objects in the database. To overcome this problem, we propose to use Bayesian estimation. The object confusion matrix, already computed in [3], can be used as an estimate of the conditional probabilities needed for this purpose. Let *Z* be the object class recognized with the global descriptor, *X* its actual class and let their sub-indexes represent each one of the potential classes (*Ball-Valve*:1, *Elbow*:2, *R-Tee*:3, *R-Socket*:4, *Butterfly-Valve*:5, *3-Way-Valve*:6), then $P(Z_{C}|X_{i})$ provides the probability of recognising an object as belonging to class $Z_{C}$ when its actual class is $X_{i}$. If C=i then it is a True Positive (TP), otherwise (C≠i) it is a False Positive (FP). Tracking the objects across the scans allows computing its class probabilities in an iterative way, selecting the one with highest probability as the recognized one.

The proposed Bayesian recognition method is shown in Algorithm 4. The observation probabilities $P(Z_{j}|X_{i})$ contained in the $P_{Z|X}$ matrix are computed from the synthetic confusion matrix (Table 3). Then, given an Object *O* and the class $Z_{C}$ resulting from the descriptor-based recognition, the next procedure is followed. If the object is observed for the first time (line 8) its prior probability is initialized considering each potential class as equi-probable (line 11). Lines 12–16 use the Bayes Theorem to compute the probability of the object belonging to each potential class *j*, given the observed class $Z_{C}$ and its prior probability O.P[j]. Finally, the most likely class is returned as the one recognised by the method.
**Algorithm 4:** Bayesian-based Recognition
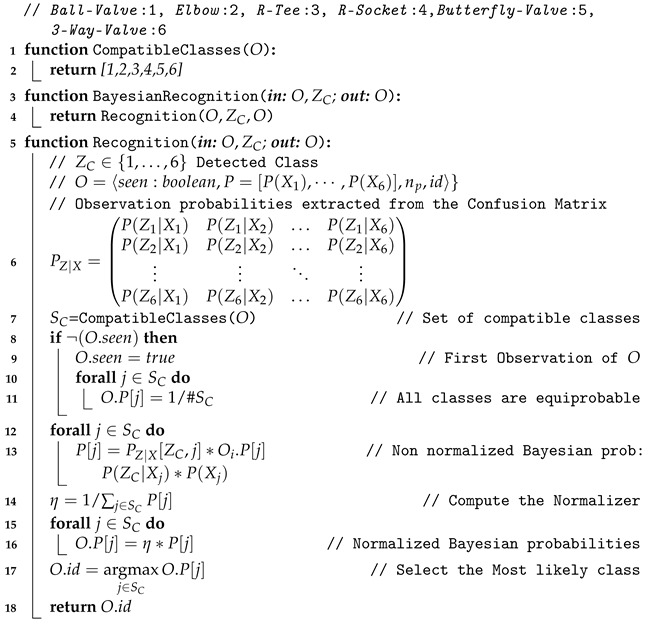


## 4. Semantic-Based Recognition

**Figure 10:**
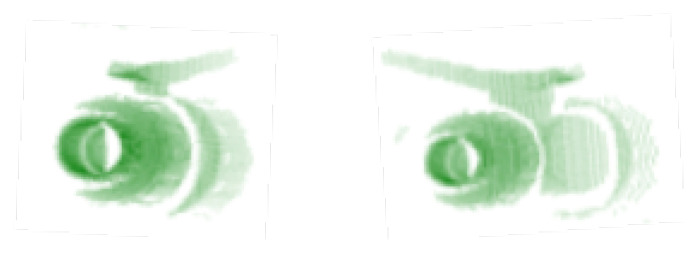
Confusing Views of the Ball-Valve and 3-Way-Valve objects.

The recognition rate can be further improved using semantic information about the number of pipes connected to the object and their geometry. This information can be used to constrain the set of potential compatible classes for a given object. As an example, if we know that an object is connected to 3 pipes, then only two candidate classes are possible—the *R-Tee* and the *3-Way-Valve*. Then, we can compute the Bayesian probabilities for these candidates classes only, assigning zero probability to the rest. Because we track the pipes to segment the objects, we can use this already available information to estimate the connectivity of the objects, and use this semantic information to improve the recognition results. The method has the potential to disambiguate confusing objects having different connectivity. For instance, certain views of the *Ball-Valve* can be easily confused with the *3-Way-Valve* (See Figure 10). This ambiguity can be easily resolved by taking into account the connectivity. Algorithm 5 shows this modification with respect to the Bayesian method algorithm discussed above. The function CompatibleClasses(O), originally returning the 6 classes, now returns only the set of classes compatible with the object connectivity geometry. It is worth noting that, given the iterative nature of the scanning process, a certain object may appear connected to a single pipe at first, and connected to two or three pipes later on. Therefore, 4 different geometric configuration may arise (Table 4):

1. Three pipes: 2 collinear and one orthogonal. This group contains the *R-Tee* and the *3-Way-Valve*.
2. Two orthogonal pipes: This group contains the *Elbow* but also the members of the previous group, since it is possible that the third pipe has not been observed yet.
3. Two collinear pipes: All objects are included in this group, except the *Elbow* (because it is orthogonal) and the R-Sockets (because only one side can be connect to a pipe of the given radius). The remaining objects admit a collinear connection to 2 pipes.
4. Single or no connection: All objects are considered as potential candidates.

**Algorithm 5:**Semantic-based Recognition
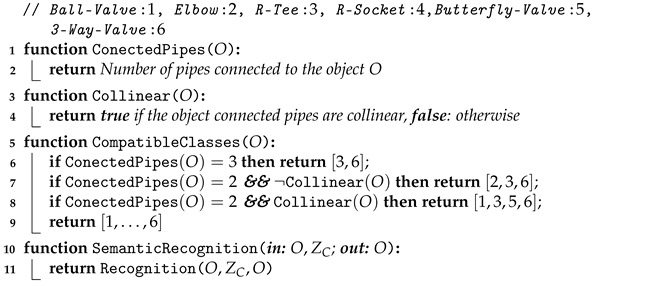


## 5. Experimental Results

### 5.1. Test Platform and Laser Scanner

Testing was conducted using the Girona 500 AUV, a lightweight intervention- and survey-capable vehicle rated for 500m depth with dimensions of 1m in height and width, and 1.5m in length. The lower hull houses the heavier elements such as the batteries and removable payload, whereas the upper hulls contain flotation material and lighter components. This arrangement enables the vehicle to be very stable in roll and pitch due to the distance between the centers of mass and flotation. The pressure sensor, the Attitude and Heading Reference System (AHRS), the Global Positioning System (GPS), the acoustic modem and the DVL provide measurements to estimate the pose of the vehicle. The current configuration of thrusters provides the AUV with 4 degrees of freedom (DoF) which can be controlled in force, velocity and position. Finally, the vehicle software architecture is integrated in Robot Operating System (ROS) [48] simplifying the systems integration.

The laser scanner was designed and developed in-house [49]. It contains a laser line projector, a moving mirror driven by a galvanometer, a camera and two flat viewports, one for the camera and one for the laser. The galvanometer is electrically synchronized with the camera, such that the image acquisition is only performed when the galvanometer is stopped, thus producing an image with only one single laser line. The sensor generates a 3D point cloud by triangulating all the laser points corresponding to the different mirror positions during a full scan. For the experiments in this paper, the scanner was configured to acquire scans at a rate of 0.5 Hz generating ≈200 k points/s and 400 lines/scan. At a nominal distance of 3 m, the distance between scan lines is ≈4.5 mm.

### 5.2. Experimental Setup

**Figure 11:**
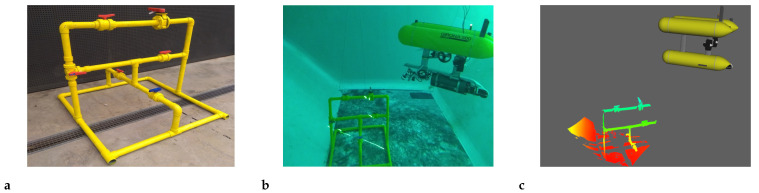
Image of the Girona 500 AUV inspecting the structure. The mapped structure before deployment (**a**), underwater view of the water tank (**b**) and online 3D visualizer with a scan of the structure (**c**).

The experiments consisted in exploring an underwater industrial structure made of pipes and valves, having approximate dimensions of 1.4 m width, 1.4 m depth and 1.2 m height (see Figure 11). During the experiment, the Girona 500 AUV was tele-operated to move around the structure. To reduce the distortions within each scan produced by the vehicle motion, the AUV was put in station-keeping mode during the acquisition of each scan. The structure was mapped at a distance ranging from 2 to 3.5 m.

During the experiment, 100 scans were processed containing a total of 523 object observations of 13 different objects from 6 different classes.

To evaluate the performance, ground truth was created by manually labelling objects appearing in the scans.

The following three object recognition methods, described in this paper, have been evaluated:

1. The Object Recognition Pipeline described in Section 2.
2. The Bayesian estimation extension presented in Section 3.
3. The Semantic Bayesian estimation extension presented in Section 4.

The three methods were tested using the two descriptors—CVFH and OUR-CVFH. These descriptors were selected because they provided the best experimental results in our previous survey paper [3].

**Figure 12:**
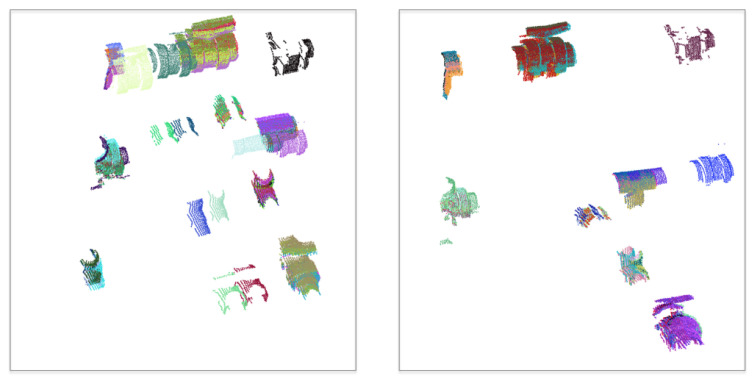
Mapped object point clouds: (**Left**) Located at their dead reckoning position; (**Right**) Located at the position estimated by the tracking using the *IJCBB* algorithm on the right.

The IJCBB method (Algorithm 3) was used to address and solve the issue of the navigation jumps, thus allowing tracking of objects across the scans. Consistent tracking of objects is required for the Bayesian estimation to work properly. The effect of the IJCBB method can be seen in Figure 12. The left side shows the accumulation of object instances using only the dead reckoning from the vehicle navigation data. The large navigation errors and the close proximity of some objects leads to some of the tracked objects being incorrectly assigned over time. The right side illustrates the improvement in the localization of these objects by using the tracking based on the IJCBB.

**Figure 13:**
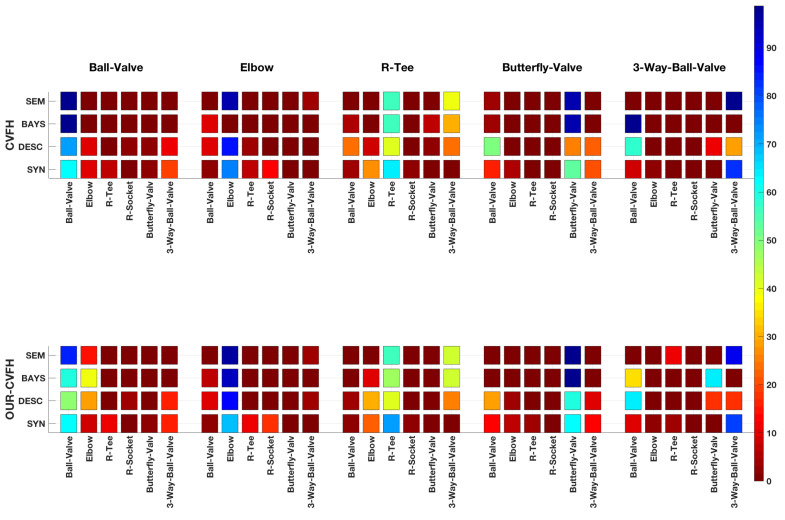
Graphical representation of the Confusion Matrices.

Figure 13 and Table 3 show the graphical and numerical representation of the confusion matrices computed for the following cases:

1. The Synthetic Confusion Matrix.
2. The Confusion Matrix based on global descriptors only.
3. The Confusion Matrix incorporating Bayesian estimation.
4. The Confusion Matrix incorporating Bayesian estimation and semantic information.

The first confusion matrix was computed based on the results of our previous paper [3]. It was obtained by averaging the confusion matrices corresponding to the partial and the global view experiments for the noise matching and resolution in the order of magnitude of the one of our scanner (σ=0.00625, resolution=0.007 [m]) and for the case where the same resolution is used for the scan and the object 3D model in the data base. The other 3 were computed from the results of the experiment.

### 5.3. Average Performance

The average object recognition rate (percentage of correctly recognized objects) for both descriptors, CVFH and OUR-CVFH, is summarized in the last column of Table 5. It can be appreciated that, as hypothesised, in both cases the Bayesian estimation improves the recognition rate achieved with the descriptor alone. Moreover, the use of semantic information further improves the results. When using the OUR-CVFH descriptor improvements (with respect to the semantic method) of 9% and 25% respectively are observed, achieving a final average recognition rate of 85%. Nevertheless, the best results are achieved using the CVFH descriptor, where the Bayesian method improves recognition by 18% and the semantic variant provides a further improvement of 21%, reaching an average recognition rate of 90%.

### 5.4. Class-by-Class Performance

**Figure 14:**
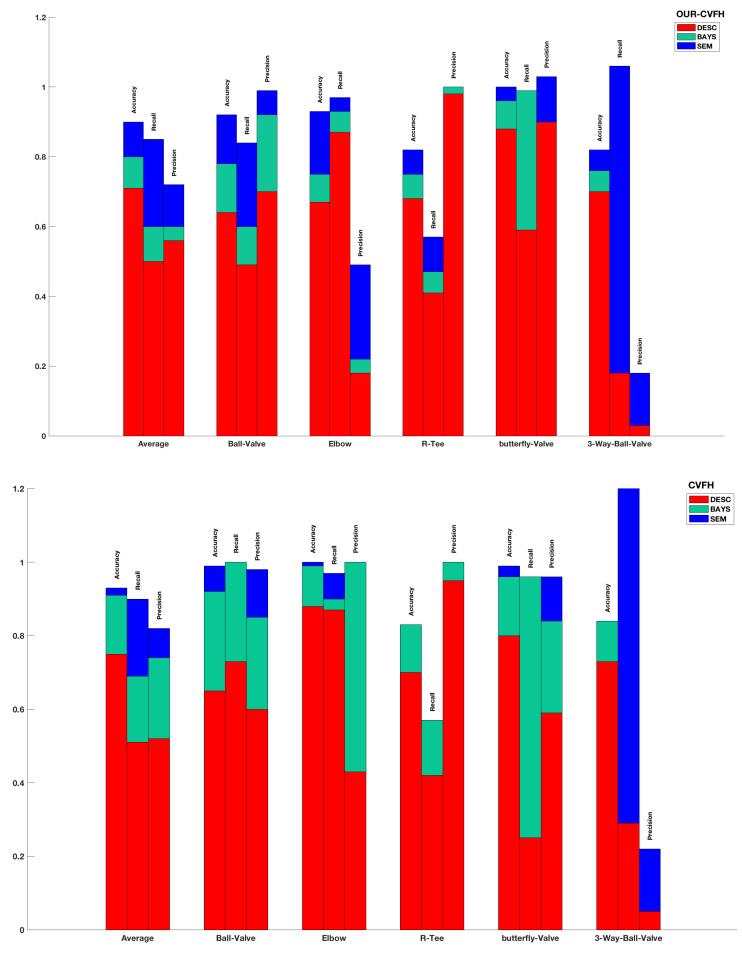
Evaluation of the recognition performance using Accuracy, Recall and Precision for descriptor-based, Bayesian-based and semantic-based method for both: (**Top**) OUR-CVFH; (**Bottom**) CVFH.

Now let us focus on the class-by-class performance. To provide a better insight, the evaluation is based on the performance metrics (recall, precision and accuracy) for each descriptor-method-class combination reported in Table 6, and illustrated graphically in Figure 14. 

#### 5.4.1. Descriptor Based Recognition Pipeline

When using only the descriptor based recognition, the performance varies across the object classes. For *CVFH*, the recall is excellent for the *Elbow*, good for the *Ball-Valve*, medium for the *R-Tee* and poor for the *Butterfly-Valve* and the *3-Way-Valve*. On the other hand, the precision is excellent for the *R-Tee*, good for the *Ball-Valve* and the *Butterfly-Valve*, medium for the *Elbow* and poor for the *3-Way-Valve*. Similar results are obtained for the OUR-CVFH descriptor which achieves an excellent recall for the *Elbow*, medium for the *Ball-Valve*, the *R-Tee* and the *Butterfly-Valve* and again bad for the *3-Way-Valve*. In this case the precision is excellent for the *R-Tee* and the *Butterfly-Valve*, good for the *Ball-Valve* and poor for the *Elbow* and the *3-Way-Valve*.

#### 5.4.2. Bayesian Estimation

When applying Bayesian estimation with the CVFH descriptor, both performance metrics improve significantly becoming excellent for the *Ball-Valve*, the *Elbow* and the *Butterfly-Valve*. For the *R-Tee* the recall is medium with an excellent precision, but for the *3-Way-Valve* both metrics are actually worse. The precision remains excellent for the *R-Tee* and improves to excellent for the *Ball-Valve*, the *Elbow* and *Butterfly-Valve*, but remains poor for the *3-Way-Valve*. For the OUR-CVFH descriptor, the performance improves slightly less. The recall remains excellent for the *Elbow* and improves to excellent for the *3-Way-Valve*. It remains good for the *R-Tee* and improves to good for the *Ball-Valve*, but still poor for the *3-Way-Valve*. On the other hand, the excellent precision of the *R-Tee* and the *Butterfly-Valve* is maintained while it evolves from good to excellent for the *Butterfly-Valve*, and from bad to poor for the *Elbow*, but remains poor for the *3-Way-Valve*. However, in general all the metrics improve.

#### 5.4.3. Bayesian Estimation and Semantic Information

When semantic information is included in the Bayesian estimation, the performance further improves. For CVFH, the recall and precision qualitative performance remains the same (mostly excellent) but their numerical values increase slightly. Moreover, the poor performance in the Bayesian estimation of the *3-Way-Valve*, improves to excellent. The OUR-CVFH descriptor improves significantly in this case. The recall, remains excellent for the *Elbow* and the *Butterfly-Valve* and improves to excellent for the *Ball-Valve* and the *3-Way-Valve* while maintaining the medium performance (but increasing by 10%) for the *R-Tee*. Its precision remains excellent for the *Ball-Valve* and *Butterfly-Valve* (in both cases increasing numerically), evolving from poor to medium for the *Elbow*, although still poor (but increasing the value) for the *3-Way-Valve*. Again, all the numerical values of the statistics improve.

The overall best results are obtained using the CVFH and the semantic-method with excellent recall and precision for every object class except the *R-Tee* which has medium recall and the *3-Way-Valve* which has poor precision.

#### 5.4.4. Discussion

Analysing the results reported in Table 4, we realise that for the *Butterfly-Valve* and the *3-Way-Valve* object classes the recall achieved with the descriptor method is significantly below the average recall. Moreover, for the *3-Way-Valve*, the Bayesian method is not improving the results but causing troubles. To understand what happens let us examine the synthetic confusion matrix (Figure 13) for both descriptors. It can be appreciated that the *Butterfly-Valve* is commonly confused with the *Ball-Valve* and the *3-Way-Valve*. For both descriptors the *Butterfly-Valve* (TP) observation probability is significantly higher (>50%) than the probabilities of the *Ball-Valve* and the *3-Way-Valve* (False Negatives (FNs)). However, when the confusion matrix is computed from the experimental data similar recognition percentages are found for OUR-CVHF while they are reversed for the CVFH, with higher probabilities for the FNs (*Ball-Valve* and *3-Way-Valve*) than for the TP (*Butterfly-Valve*). Using the partial views observed with the scanner in the experiment, CVFH is not working as well as it did with the synthetic ones simulated in [3]. Instead, the experimental and synthetic behaviours of OUR-CVFH are closer.

**Figure 15:**
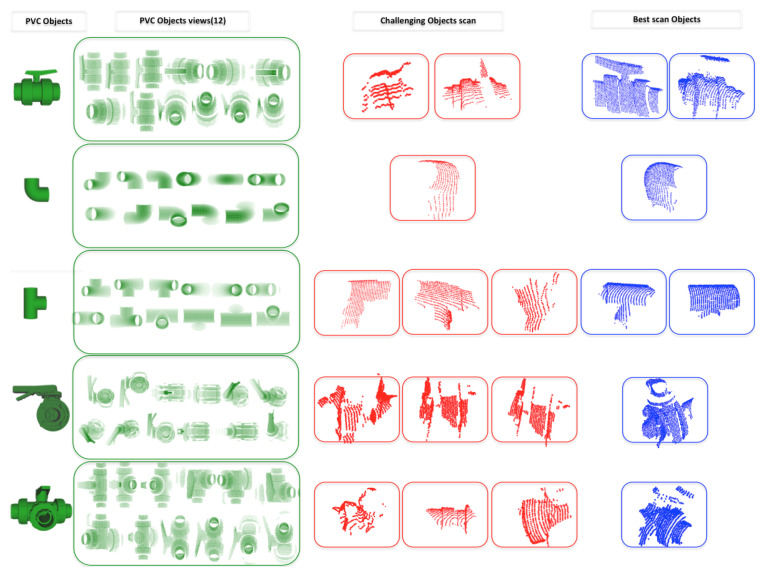
PVC objects used in the experiment (first column) with their respective database views (second column). The last two columns provide manually selected examples of segmented objects from the experiments, with the most difficult in red and the easiest in blue.

The problem is more severe with the *3-Way-Valve* whose experimental and synthetic recognition percentages are also reversed, and in addition suffering a poor accuracy, indicating that most of the observations are actually FNs. If we take a close look at the partial views obtained after the segmentation (see Figure 15) we can see that unfortunately most of them correspond to challenging scans (in red). Recognizing the object from those views is difficult, if not unfeasible, even for the human perception. This suggests that a method should be designed to decide which view is representative and therefore worth attempting to recognize and which one should just be ignored.

For the *Butterfly-Valve*, the Bayesian method does an excellent job, bringing the recall and the precision to 0.96 and 0.84 for CVFH and to 0.99 and 0.87 for OUR-CVFH. To understand why, let us focus on the CVFH descriptor. The TP probability ($P(Z_{5}|X_{5})=0.54$) is discriminant in comparison to the FP probabilities ($P(Z_{5}|X_{1})=0.02$, $P(Z_{5}|X_{2})=0.01$, $P(Z_{5}|X_{3})=0.01$, $P(Z_{5}|X_{4})=0.1$, $P(Z_{5}|X_{6})=0.01$).This means that a single TP observation assigns more weight to the probability of the TP-class than several FP observations do with their counterparts. Its accuracy (0.59) also helps, since it means that there are more TPs than FPs, driving therefore, the Bayesian estimation towards the correct class. The same happens for OUR-CVFH where we start from a much better point with a recall of 0.59 and a good precision of 0.9. Unfortunately this is not the case for the *3-Way-Valve* whose performance even decreases for both descriptors when using the Bayesian method. For the CVFH case, even though the TP probability ($P(Z_{6}|X_{6})=0.84$) is high, there are two significant FP probabilities in play ($P(Z_{6}|X_{1})=0.19$, $P(Z_{6}|X_{5})=0.21$).Adding this to the very high number of FPs (where precision is only 5%) explains the fact that the Bayesian method is not helping but actually making it worse. It is worth remembering that the origin of the problem is the fact that the *3-Way-Valve* partial views obtained after the segmentation are poor representatives of the object class.

When semantics are taken into account during the Bayesian estimation process, the results improve further. Now, Bayesian estimation only affects those classes which are compatible in terms of pipe connectivity. Because classes having a significant confusion, like the *3-Way-Valve* and the *Ball-Valve* for instance, have different connectivity (3 and 2 respectively), so they can be easily distinguished by the number of connected pipes. This further improves the results of all the object classes, recovering, in particular the recall of the *3-Way-Valve*. However, the precision is still poor because there are a significant number of FPs which are compatible in terms of connectivity. This is the case of the *R-Tee* class, which is often confused with the *3-Way-Valve*. Because both classes are equivalent in terms of connectivity, the semantic-based method is not able to help. Again, it is worth noting that the origin of the problem is the poor *3-Way-Valve* views observed in the experiment.

## 6. Conclusions

Detecting and recognizing multiply connected objects in underwater environments is a complex task that must be performed under the constraints of the sensor, the acquisition platform and the nature of the shapes of the objects we wish to detect. In this paper, we have presented a method to recognize 3D objects as part of a pipeline for acquiring and processing non-colored point clouds using point features. The presented method is intended to be used for Inspection, Maintenance and Repair (IMR) of industrial underwater structures. As a representative example for testing, the developed methods were applied to a test structure consisting of pipes and connected PVC objects. These objects pose considerable challenges for an object recognition system, due to view-dependant similarities in their appearance. As such, the testing conditions capture the main difficulties of a real scenario for underwater Inspection, Maintenance and Repair (IMR).

An initial goal of this paper was to develop methods for the pre-processing of point cloud data that would potentiate and facilitate the recognition task. These methods include plane and pipe detection, semantic segmentation, and object tracking based on the IJCBB algorithm. Semantic segmentation aimed at better obtaining a set of points that belong to the objects, in order to reduce the negative impact of the presence of parts of the pipes, during recognition. The semantic segmentation involved determining the pipe intersections, to then allow for computing candidate object locations and therefore perform a better crop of the input scan so that it tightly encapsulates the object to be recognized. The IJCBB-based tracking aimed at correcting the effects of inconsistencies in the robot navigation, which appeared in the form of sudden jumps in the estimated pose of the AUV that preclude the tracking of the objects along scans.

The second goal, which conveys the most important contributions of this study, is the comparison of three established methods, namely descriptor-based, Bayesian-based and semantic-based recognition.

The descriptor-based method, which was used in our previous work [3] to detect individual objects attained good performance, especially when the scans contained a complete, occlusion-free view of the objects. Considerably better results were obtained by tracking objects along scans and using a Bayesian framework to keep recognition probabilities assigned to each object, achieving, for the CVFH descriptor an 18% increase in the average recognition rate.

It should be noted that there is a significant increase in the recognition rate when the object to be detected satisfies the conditions that a relevant part of the object shape is present, and that distinctive features of the objects are visible. Clear examples where these conditions were not met were the *Butterfly-valve* and the *3-way-ball-valve*. These two objects were affected by poorly segmented views, which resulted in the loss of the distinctive features needed for discrimination among objects. In this case, the distinctive features are the handle for the *Butterfly-valve* and the part of the opening of the *3-way-ball-valve*.

These problems have been addressed by semantics-based recognition, which considers a set of rules based on pipe intersections that allow computation and updating of the Bayesian estimation approach, considering only objects that verify these rules. For the CVFH descriptor, the inclusion of semantic rules increases the average recognition rate by 21% with respect to the Bayesian method.

## 7. Future Work

Although there are clear advantages to using semantic information with the Bayesian method for recognition, the dependence of the recognition system on the segmented views makes it vulnerable in some cases. Motivated by the improved results achieved by using semantic information within the Bayesian approach, near-term future work will concentrate on the integration of the approach within a Simultaneous Localization And Mapping (SLAM) framework. Among other advantages, such a framework will further facilitate the association of observations of objects, releasing the constraint of needing sufficient temporal overlap between scans, which is implicitly required in the tracking process. Moreover, SLAM will provide a consistent long term drift-less navigation, allowing to explore the structure from different viewpoints. This will enrich the set of views used during the Bayesian recognition providing more robust results.

From the experiments with the database views generated from the CAD models, we concluded that significant perceptual differences were observed between the rendered views in the database and the real views captured by the laser scanner. Such differences impact the recognition performance negatively. This problem will be addressed, in the near future, by collecting database views with the laser scanner used in the tank during the experiment.

As longer term future work, the approach will be used as a building block towards a complete system for autonomous intervention by I-AUVs working in industrial underwater scenarios.

## Figures and Tables

**Table 1:** Polyvinylchloride (PVC) pressure pipes objects used in the experiments.

PVC Objects	Id Name	Size (mm${}^{3}$)	PVC Objects Views (12)
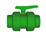	1-Ball-Valve	198×160×120	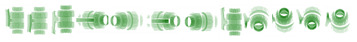
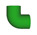	2- Elbow	122.5×122.5×77	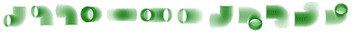
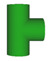	3- R-Tee	122.5×168×77	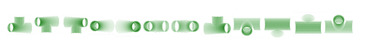
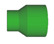	4- R-Socket	88×75×75	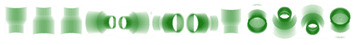
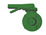	5- Butterfly-Valve	287.5×243×121	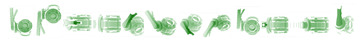
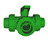	6- 3-Way-Ball-Valve	240×160×172	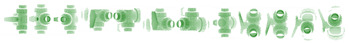

**Table 2:** Summarized characteristics of the two descriptors used in this paper, respectively CVFH and OUR-CVFH. The “based on” column indicates if the descriptor evolved directly from another approach. The “use of normals” indicates whether the method uses surface normals for computing the descriptor, while the last column indicates the length of the descriptor vector.

Descriptor	Main Characteristics		
	Based on	Use of Normals	Descriptor Size
Clustered Viewpoint Feature Histogram (CVFH)-2011—[42]	Viewpoint Feature Histogram(VFH) [46]	Yes	308
Oriented, Unique and Repeatable CVFH (OUR-CVFH)-2012—[43]	CVFH [42]	Yes	308

**Table 3:** Confusion Matrices expressed as a numerical %.

Objects																															
Descriptors	Experiment	**Ball Valve**						**Elbow**						**R-Tee**						**Butterfly-Valve**						**3-Way-Ball-Valve**					
		**1**	**2**	**3**	**4**	**5**	**6**	**1**	**2**	**3**	**4**	**5**	**6**	**1**	**2**	**3**	**4**	**5**	**6**	**1**	**2**	**3**	**4**	**5**	**6**	**1**	**2**	**3**	**4**	**5**	**6**
**CVFH**	**SYN**	63	10	7	1	2	19	2	75	7	14	1	1	4	27	65	2	1	1	17	5	1	1	54	21	9	3	1	1	1	84
	**DESC**	72.5	9.5	1	2.5	3	11.5	10	86.67	3.33	0	0	0	23.5	8	41.5	0	2.5	24.5	50.67	0	1.33	0	25.33	22.67	58.82	0	0	0	11.76	29.41
	**BAYS**	100	0	0	0	0	0	10	90	0	0	0	0	5.5	0	57	0	7	30.5	4	0	0	0	96	0	100	0	0	0	0	0
	**SEM**	100	0	0	0	0	0	0	96.67	0	0	0	3.33	1	0	57	0	1.5	40.5	4	0	0	0	96	0	0	0	0	0	0	100
**OUR-CVFH**	**SYN**	62	8	11	1	2	16	2	68	11	17	1	1	2	22	71	3	1	1	13	7	4	1	63	13	10	3	4	1	1	81
	**DESC**	49	28	1	4	1	16	10	86.67	0	0	0	3.33	3	30	40	0	0	26	28	4	0	0	58.67	9.33	64.71	0	0	0	17.65	17.65
	**BAYS**	60	40	0	0	0	0	6.67	93.33	0	0	0	0	1	10.5	46.5	0	0	42	0	0	0	0	98.67	1.33	35.29	0	0	0	64.71	0
	**SEM**	84	15	1	0	0	0	0	96.67	0	0	0	3.33	1	0	57	0	0	42	0	0	0	0	98.67	1.33	0	0	11.76	0	0	88.24

**Table 4:** Semantic connection of Objects.

Type of Connection	Pipes Disposition			Potential Objects Candidate
	$n_{p}$	=	⊥	
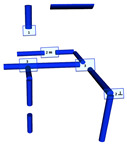	3	2	1	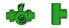
	2	0	2	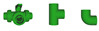
	2	2	0	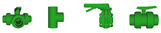
	1|0	1|0	1|0	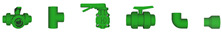

**Table 5:** Average of recognition per Object and methods for all descriptors, represented in a table.

Descriptors	Experiment	Average
CVFH	Descriptor	51.2
	Bayesian	68.6
	Semantic	90
OUR-CVFH	Descriptor	50.8
	Bayesian	59.8
	Semantic	85

**Table 6:** Assessment of the recognition performance through Accuracy, Recall and Precision. Qualitative labels used in the text: bad (0–0.2); poor (0.2–0.4); medium; good; excellent.

Objects																			
Descriptors	Experiment	Ball Valve			Elbow			R-Tee			Butterfly-Valve			3-Way-Ball-Valve			Average		
		Accuracy	Recall	Precision	Accuracy	Recall	Precision	Accuracy	Recall	Precision	Accuracy	Recall	Precision	Accuracy	Recall	Precision	Accuracy	Recall	Precision
**CVFH**	**DESC**	0.65	0.73	0.60	0.88	0.87	0.43	0.70	0.42	0.95	0.80	0.25	0.59	0.73	0.29	0.05	0.75	0.51	0.52
	**BAYS**	0.92	1.00	0.85	0.99	0.90	1.00	0.83	0.57	1.00	0.96	0.96	0.84	0.84	0.00	0.00	0.91	0.69	0.74
	**SEM**	0.99	1.00	0.98	1.00	0.97	1.00	0.83	0.57	1.00	0.99	0.96	0.96	0.84	1.00	0.17	0.93	0.90	0.82
**OUR-CVFH**	**DESC**	0.64	0.49	0.70	0.67	0.87	0.18	0.68	0.41	0.98	0.88	0.59	0.90	0.70	0.18	0.03	0.71	0.50	0.56
	**BAYS**	0.78	0.60	0.92	0.75	0.93	0.22	0.75	0.47	1.00	0.96	0.99	0.87	0.76	0.00	0.00	0.80	0.60	0.60
	**SEM**	0.92	0.84	0.99	0.93	0.97	0.49	0.82	0.57	0.97	1.00	0.99	1.00	0.82	0.88	0.15	0.90	0.85	0.72

## Data Availability

Data sharing is not applicable to this article as no new data were created in this study.
